# Supplementary figures and images for: The extracellular matrix supports breast cancer cell growth under amino acid starvation by promoting tyrosine catabolism
Source: PLoS Biol. 2024 Jan 16;22(1):e3002406. doi: 10.1371/journal.pbio.3002406 (PMC10791009; doi:10.1371/journal.pbio.3002406)

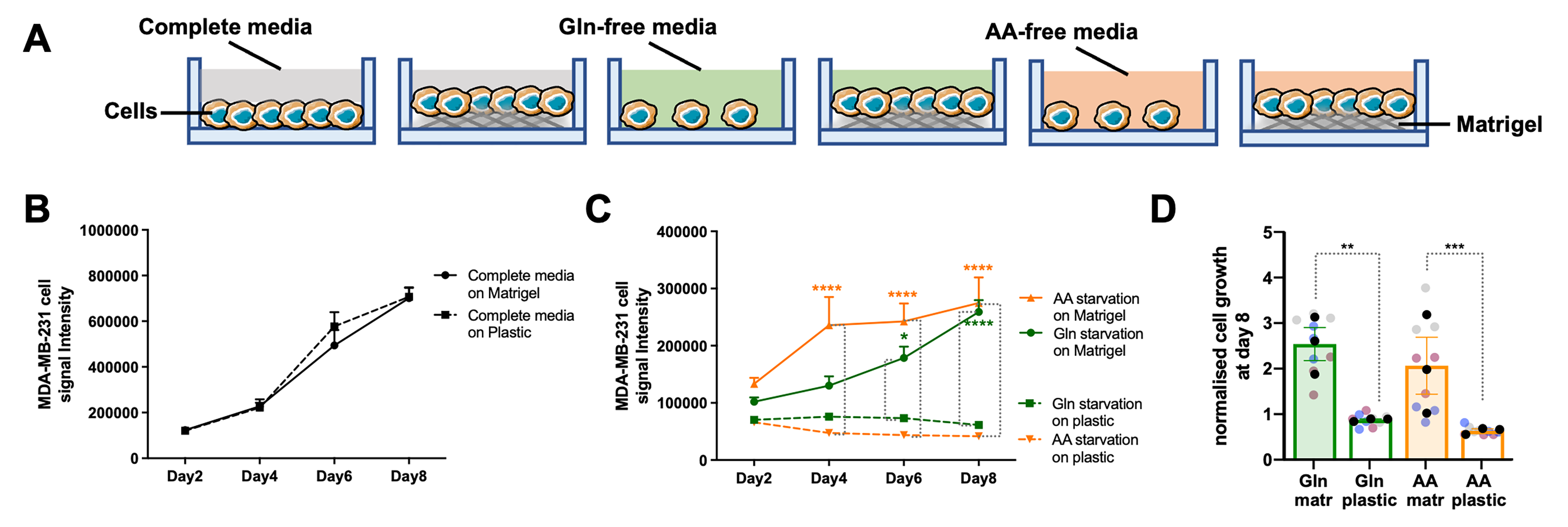

Supplement: S1 Fig — (A) Schematic, cell proliferation experiments. (B–D) MDA-MB-231 cells were seeded on plastic or 3 mg/ml Matrigel (matr) for 8 days under complete media, glutamine (Gln) or amino acid (AA) starvation, fixed, stained with DRAQ5 and imaged with a Licor Odyssey system. Signal intensity was calculated by Image Studio Lite software. Values are mean ± SEM from 3 independent experiments (the black dots in the bar graph represent the mean of individual experiments). *p < 0.05, **p < 0.001, ****p < 0.0001. (C) Two-way ANOVA, Tukey’s multiple comparisons test; (D) Kruskal–Wallis, Dunn’s multiple comparisons test. All the raw data associated with this figure are available in S9 Data. (TIF) [file pbio.3002406.s001.tif]

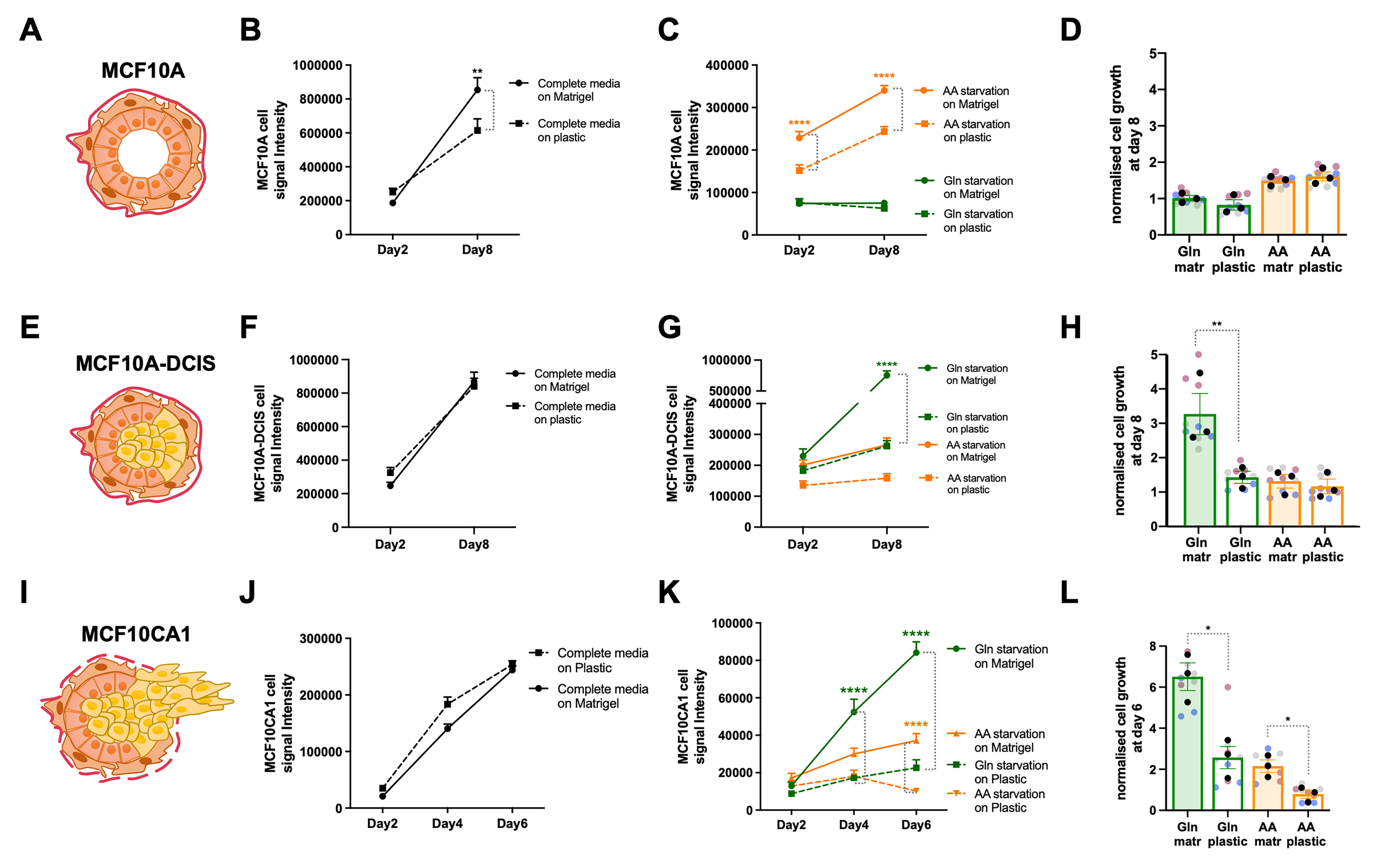

Supplement: S2 Fig — MCF10A (A–D), MCF10DCIS (E–H) and MCF10CA1 (I–L) cells were seeded on plastic or 3 mg/ml Matrigel for 6 or 8 days under complete media, glutamine (Gln) or amino acid (AA) starvation, fixed, stained with DRAQ5 and imaged with a Licor Odyssey system. Signal intensity was calculated by Image Studio Lite software. Values are mean ± SEM from 3 independent experiments (the black dots in the bar graphs represent the mean of individual experiments). *p < 0.05, **p < 0.01, ****p < 0.0001 (B, C, G, K) two-way ANOVA, Tukey’s multiple comparisons test. (H, L) Kruskal–Wallis, Dunn’s multiple comparisons test. All the raw data associated with this figure are available in S10 Data. (TIF) [file pbio.3002406.s002.tif]

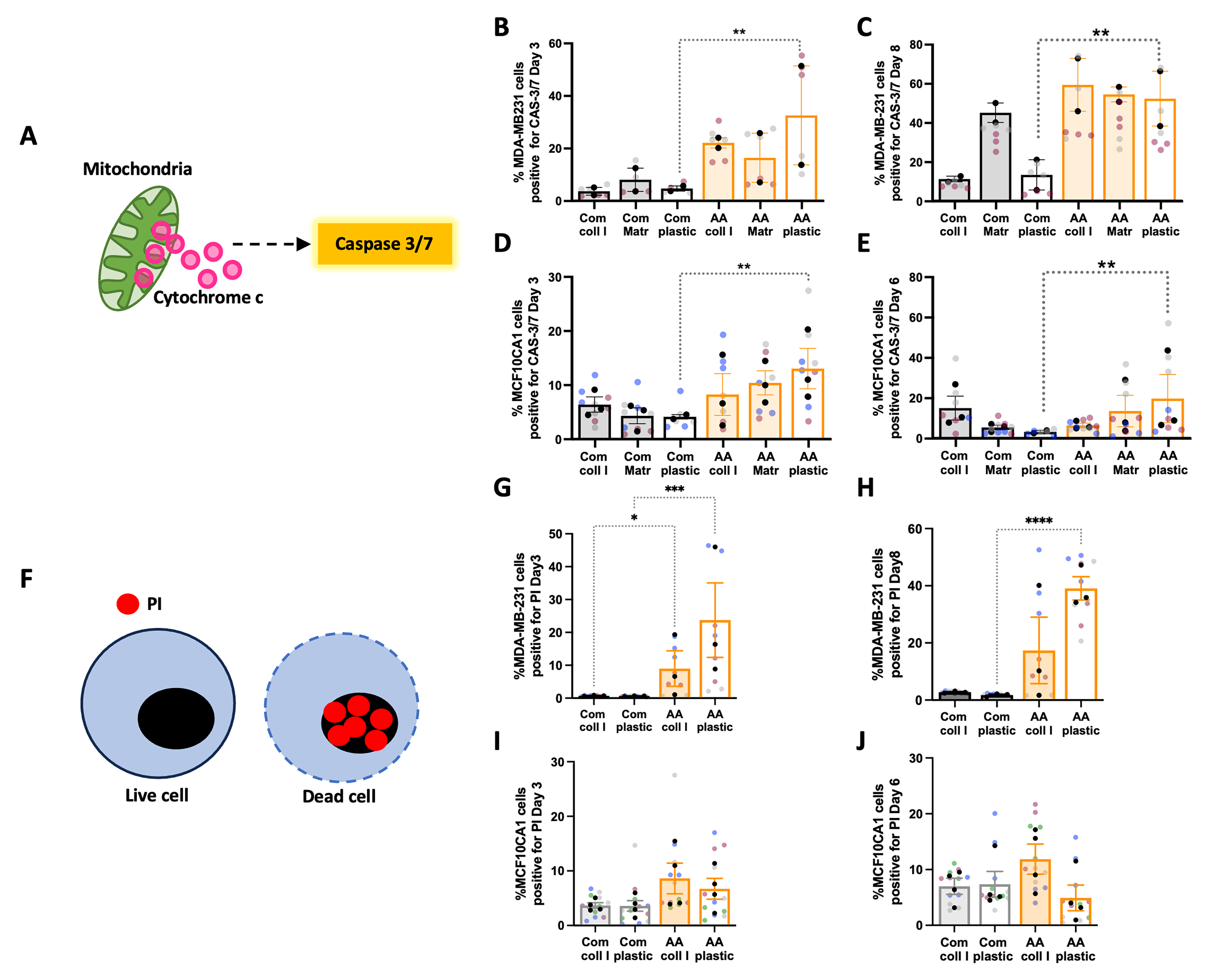

Supplement: S3 Fig — (A, F) Schematic, caspase3/7 activation and propidium iodide (PI) staining. MDA-MB-231 cells were seeded on plastic, 2 mg/ml collagen I (coll I) or 3 mg/ml Matrigel (Matr) for (B, G) 3 or (C, H) 8 days in complete media (Com) or amino acid-free media (AA). MCF10CA1 cells were seeded on plastic, 2 mg/ml collagen I (coll I) or 3 mg/ml Matrigel (Matr) for (D, I) 3 or (E, J) 6 days in complete media (Com) or amino acid-free media (AA). Cells were fixed and stained for activated caspase-3/7 (CAS-3/7, B–E) or PI (G–J). Images were collected by ImageXpress micro and analysed by CME software. Values are mean ± SEM of at least 6 replicates from at least 2 independent experiments (the black dots represent the mean of individual experiments). *p < 0.05, **p < 0.01, ***p < 0.001, ****p < 0.0001 Kruskal–Wallis, Dunn’s multiple comparisons test. All the raw data associated with this figure are available in S11 Data. (TIF) [file pbio.3002406.s003.tif]

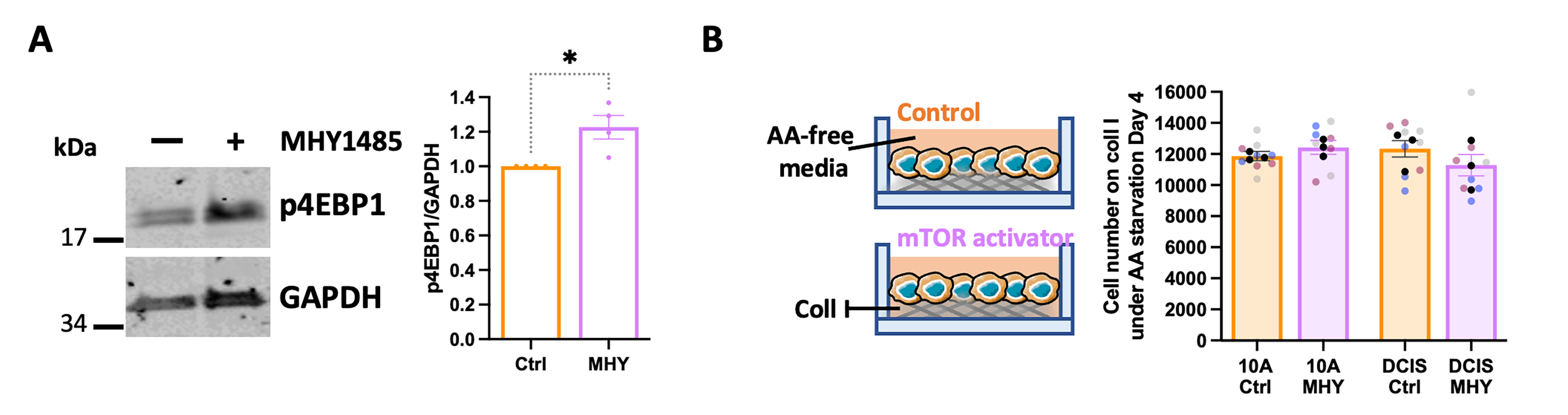

Supplement: S4 Fig — (A) MCF10A cells were amino acid starved and treated with 5 μm MHY1485 (+) or vehicle control (-) for 24 h, lysed and p-4EBP1 and GAPDH levels were assessed by western blotting. Values are mean ± SEM from 4 independent experiments; *p < 0.05 Mann–Whitney test. (B) MCF10A (10A) and MCF10A-DCIS (DCIS) cells were grown on 2 mg/ml collagen I for 4 days under amino acid (AA) starvation, in the presence of 5 μm MHY1485 (MHY) or vehicle control (Ctrl). Cells were fixed, stained with Hoechst 33342, imaged by ImageXpress micro and analysed by MetaXpress software. Values are mean ± SEM from 3 independent experiments (the black dots represent the mean of individual experiments). All the raw data associated with this figure are available in S12 Data. (TIF) [file pbio.3002406.s004.tif]

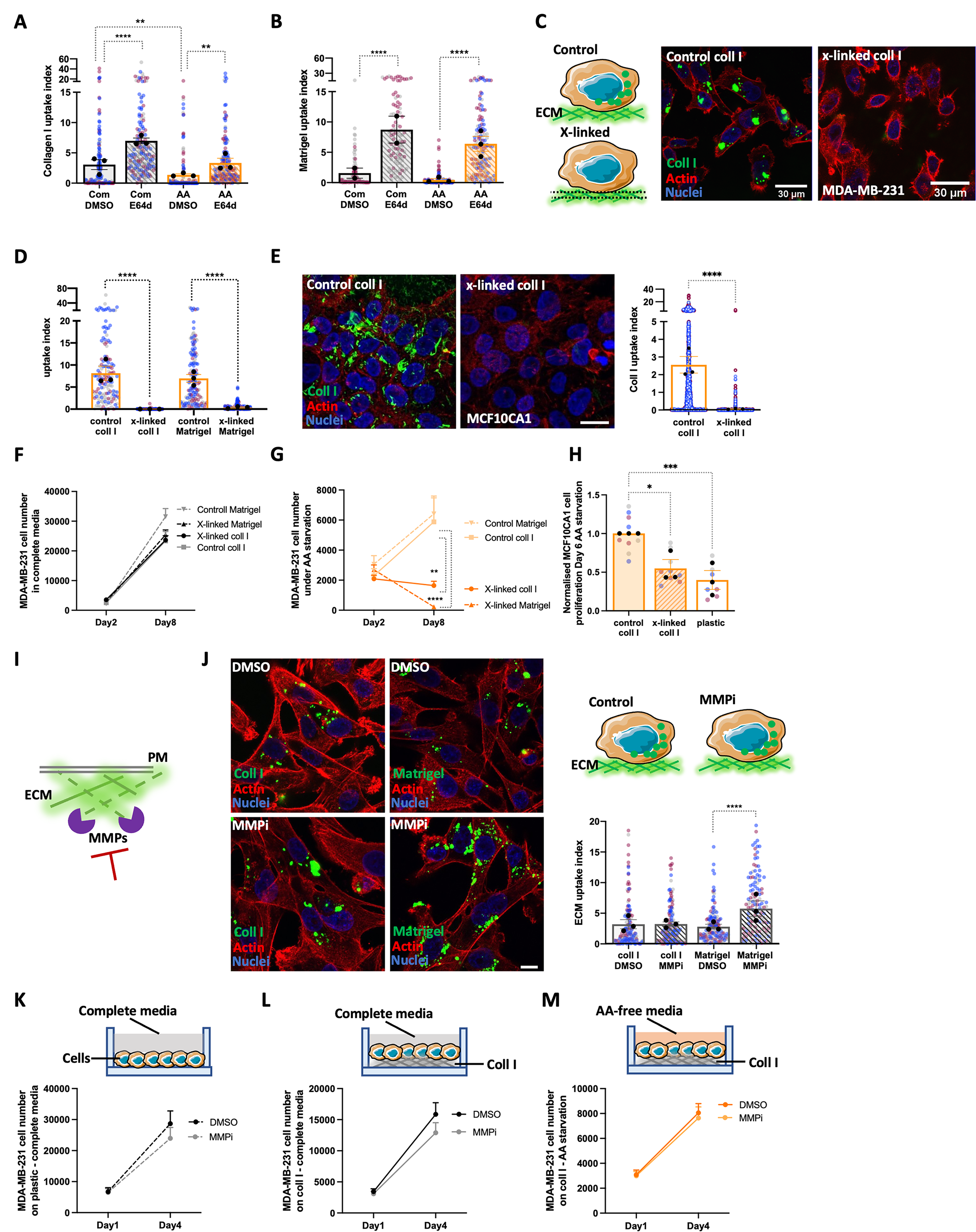

Supplement: S5 Fig — MDA-MB-231 cells were plated under complete (Com) or amino acid depleted (AA) media on NHS-fluorescein labelled (A) 2 mg/ml collagen I (coll I) or (B) 3 mg/ml Matrigel-coated dishes for 3 days, in the presence of the lysosomal inhibitor E64d (20 μm) or DMSO (control). (C–E) 2 mg/ml collagen I (coll I) or 3 mg/ml Matrigel-coated dishes were labelled with NHS-Fluorescein (green) and treated with 10% glutaraldehyde for 30 min or left untreated. MDA-MB-231 (C, D) or MCF10CA1 (E) cells were plated on the cross-liked (x-linked) matrices for 3 days under amino acid (AA) starvation in the presence of E64d (20 μm). Cells were fixed and stained for actin (red) and nuclei (blue). Samples were imaged with a Nikon A1 confocal microscope and ECM uptake index was calculated with image J. Bar, 30 μm (C) and 20 μm (E). More than 150 cells per condition in 2 or 3 independent experiments were analysed, the black dots represent the mean of individual experiments. **p < 0.01, ****p < 0.0001 Kruskal–Wallis, Dunn’s multiple comparisons test. MDA-MB-231 (F, G) and MCF10CA1 (H) cells were seeded on untreated (control) or cross-linked (X-linked) 2 mg/ml collagen I (coll I) or 3 mg/ml Matrigel under complete media (com, (F) or amino acid (AA) starvation (G, H) for 6 or 8 days). Cells were fixed, stained with Hoechst 33342, imaged by ImageXpress micro and analysed by MetaXpress software. *p < 0.05, ***p < 0.001, ****p < 0.0001 two-way ANOVA, Tukey’s multiple comparisons (G); Kruskal–Wallis, Dunn’s multiple comparisons test (H). (I) Schematic, MMP activity. (J) MDA-MB-231 cells were plated in complete media on NHS-fluorescein labelled 2 mg/ml collagen I (coll I) or 3 mg/ml Matrigel-coated dishes for 3 days, in the presence of 20 μm E64d and 10 μm GM6001 (MMPi) or DMSO (control). Cells were imaged and quantified as in (C). Bar, 10 μm. About 150 cells per condition from 3 independent experiments were analysed, the black dots represent the mean of individual experiments. ****p < 0.0001 Kruskal–Wal [file pbio.3002406.s005.tif]

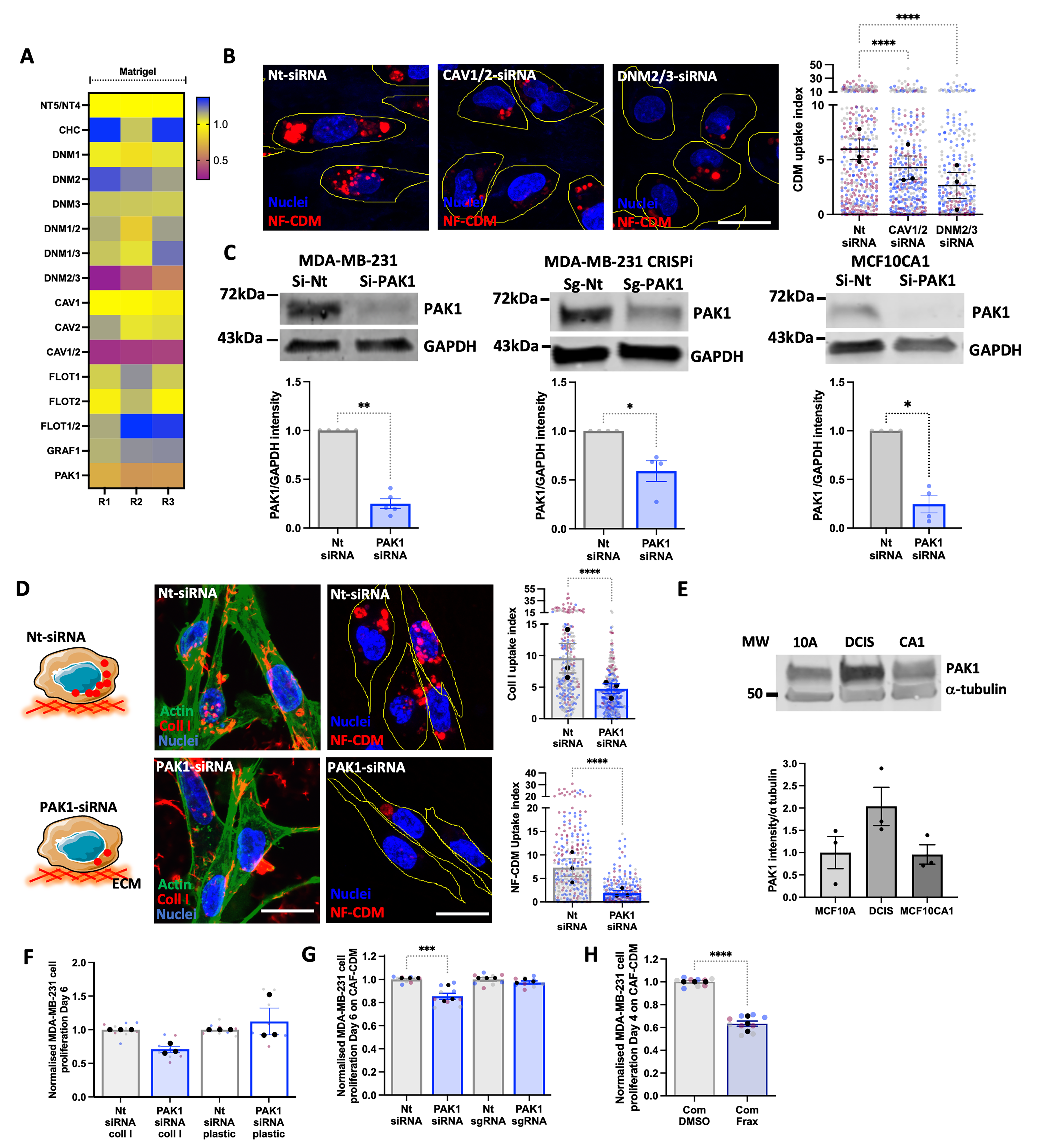

Supplement: S6 Fig — MDA-MB-231 cells were transfected with siRNA targeting the indicated genes, seeded on pH-rodo labelled 0.5 mg/ml Matrigel (A) or NF-CDM (B) for 6 h, stained with Hoechst 33342, imaged live with an Opera Phenix microscope, and analysed with Columbus software (A) or imaged live with a Nikon A1 confocal microscope and quantified with Image J. Scale bar, 20 μm (B). (C) MDA-MB-231, MDA-MB-231 CRISPRi, and MCF10CA1 cells were transfected with an siRNA targeting PAK1 (PAK1-siRNA), a non-targeting siRNA control (nt-siRNA), a synthetic guide RNA targeting PAK1 (PAK1-sgRNA) or a non-targeting synthetic guide RNA control (nt-sgRNA), lysed and PAK1 and GAPDH expression were measured by western blotting. (D) MDA-MB-231 cells were transfected with an siRNA targeting PAK1 (PAK1-siRNA) or a non-targeting siRNA control (nt-siRNA), plated on pH-rodo labelled NF-CDM (red) or Alexa Fluor 555 labelled 1 mg/ml collagen I (coll I, red) for 6 h, stained with Hoechst 33342 (blue) and imaged live with a Nikon A1 confocal microscope or fixed and stained for actin (green) and nuclei (blue). Scale bar, 20 μm. ECM uptake index was quantified with Image J. (E) MCF10A (10A), MCF10A-DCIS (DCIS), and MCF10CA1 (CA1) were lysed and PAK1 and GAPDH expression were measured by western blotting. MDA-MB-231 (F, G) and MDA-MB-231 CRISPRi (G) cells were plated on 2 mg/ml collagen I (coll I, F) or CAF-CDM (G), transfected with an siRNA targeting PAK1 (PAK1-siRNA), a non-targeting siRNA control (nt-siRNA, F, G), a synthetic guide RNA targeting PAK1 (PAK1-sgRNA) and a non-targeting synthetic guide RNA control (nt-sgRNA, G) and cultured in complete media for 6 days. Cells were fixed and stained with Hoechst 33342. MDA-MB-231 cells (H) were grown on CAF-CDM in complete media for 4 days in the presence of 3 μm FRAX597, fixed and stained with Hoechst 33342. Images were collected by ImageXpress micro and analysed by MetaXpress software. Values are mean ± SEM from at least 3 independent experiments. *p < 0.05, **p < [file pbio.3002406.s006.tif]

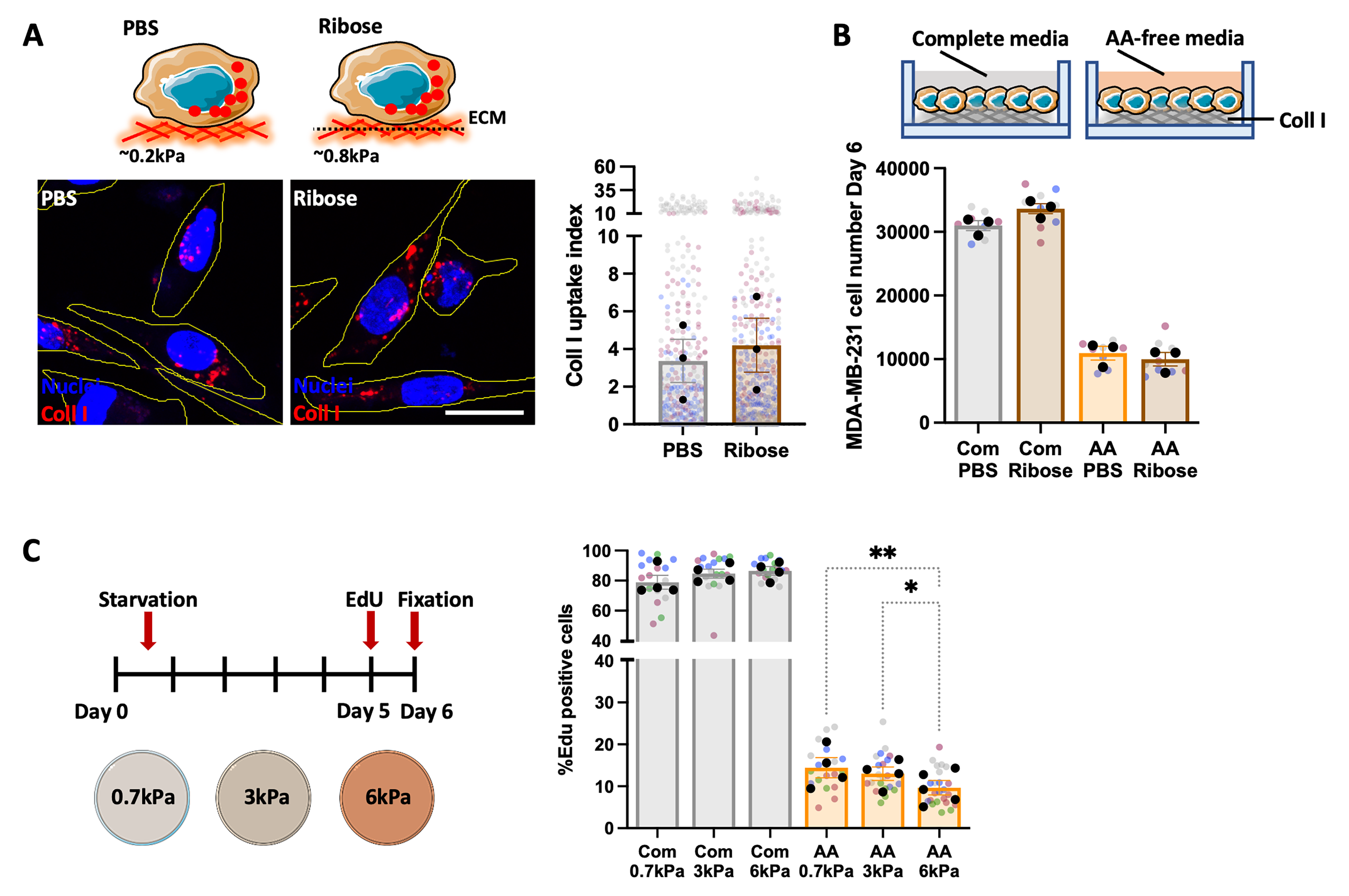

Supplement: S7 Fig — (A) 1 mg/ml collagen I gels were treated with 200 mM ribose for 48 h or left untreated and labelled with pH-rodo. MDA-MB-231 cells were seeded for 6 h, stained with Hoechst 33342, imaged live with a Nikon A1 confocal microscope and analysed with Image J. Bar, 20 μm. Values are mean ± SEM from 3 independent experiments. (B) MDA-MB-231 cells were plated on 200 mM ribose-treated or control collagen I, cultured in complete (Com) or amino acid-free (AA) media for 6 days, fixed and stained with Hoechst 33342. Images were collected by ImageXpress micro and analysed by MetaXpress software. Values are mean ± SEM from 3 independent experiments. (C) MDA-MB-231 cells were plated on polyacrylamide hydrogels of the indicated stiffness coated with 1 mg/ml collagen I, cultured in complete (Com) or amino acid-free (AA) media for 5 days, incubated with EdU for 1 day, fixed and stained with Hoechst 33342 and Click iT EdU imaging kit. The percentage of EdU positive cells was measured with Image J. Values are mean ± SEM from 3 independent experiments. *p = 0.0427, **p = 0.0044, Kruskal–Wallis, Dunn’s multiple comparisons test. Parts of the figure were drawn by using pictures from Servier Medical Art. Servier Medical Art by Servier is licensed under a Creative Commons Attribution 3.0 Unported License (https://creativecommons.org/licenses/by/3.0/). All the raw data associated with this figure are available in S15 Data. (TIF) [file pbio.3002406.s007.tif]

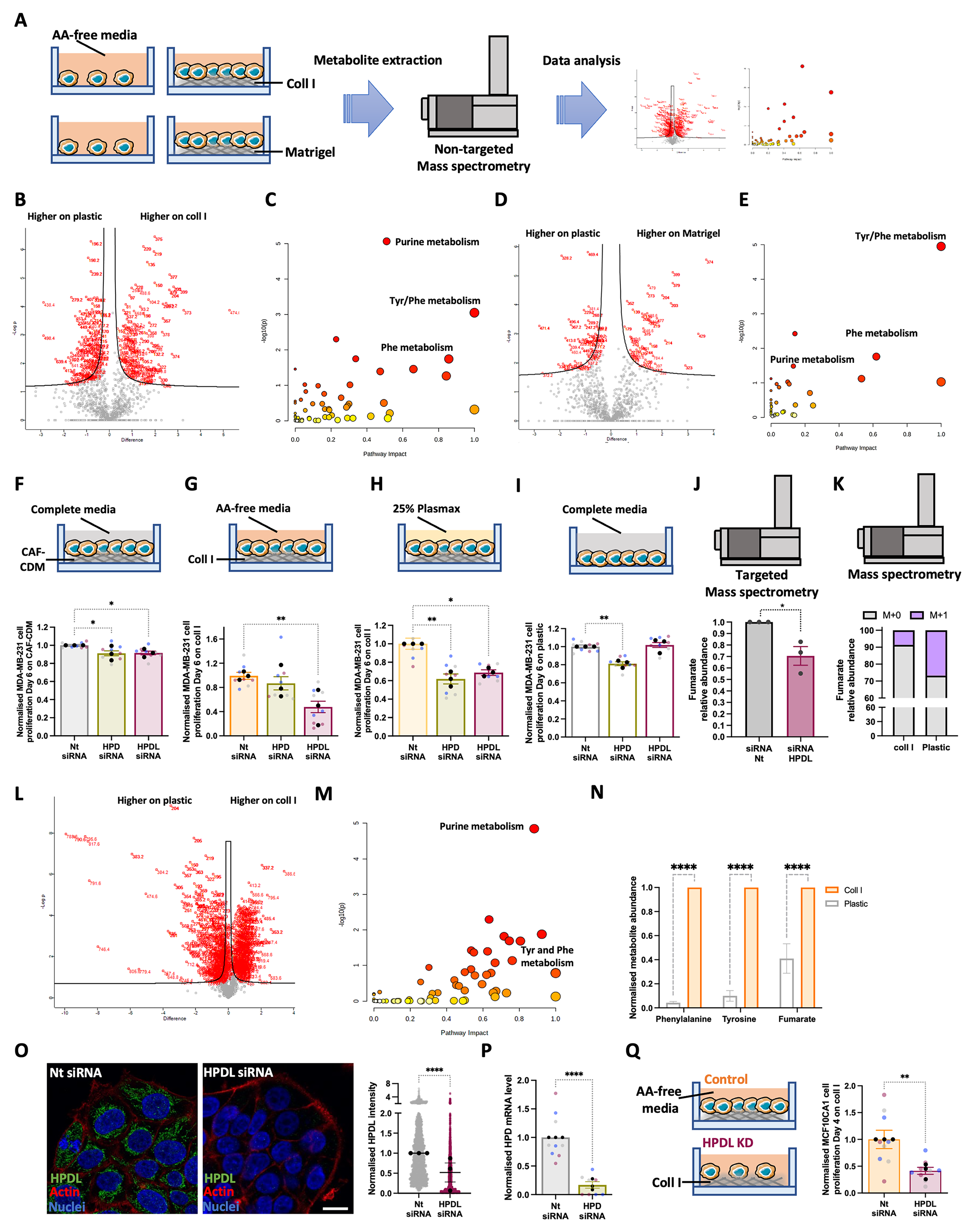

Supplement: S8 Fig — MDA-MB-231 (A–E) and MCF10CA1 (L, M) cells were plated on plastic, 2 mg/ml collagen I or 3 mg/ml Matrigel for 6 days in amino acid-free media. Metabolites were extracted and quantified by non-targeted mass spectrometry. Volcano plots (B, D, L) and enriched metabolic pathways (C, E, M) are presented. These datasets are deposited in ORDA (https://doi.org/10.15131/shef.data.21608490). (F–I) MDA-MB-231 or MCF10CA1 (Q) cells were plated on CAF-CDM (F), 2 mg/ml collagen I (coll I, G, H, Q) or plastic (I), transfected with siRNA targeting HPD (HPD siRNA), HPDL (HPDL siRNA) or non-targeting siRNA control (Nt siRNA) and cultured in complete, 25% Plasmax or amino acid-free media for 6 days. Cells were fixed and stained with Hoechst 33342. Images were collected by ImageXpress micro and analysed by MetaXpress software. Values are mean ± SEM and from 3 independent experiments. *p < 0.05, **p < 0.01, ****p < 0.0001 Kruskal–Wallis, Dunn’s multiple comparisons test. (J) MDA-MB-231 cells were plated on 2 mg/ml collagen I, transfected with siRNA targeting HPDL (HPDL siRNA) or non-targeting siRNA control (Nt siRNA) and cultured in amino acid depleted media for 3 days. Metabolites were extracted and fumarate was measured by targeted mass spectrometry. *p = 0.0126 Mann–Whitney test. (K) MDA-MB-231 cells were grown in amino acid-free media in the presence of C13 Tyrosine for 6 days. Metabolites were extracted and fumarate isotopologue abundance was quantified by targeted mass spectrometry. (N) Metabolites were prepared as in (A) and the levels of phenylalanine, tyrosine, and fumarate were measured by targeted mass spectrometry. (O) MCF10CA1 cells transfected with siRNA targeting HPDL (HPDL siRNA) or non-targeting siRNA control (Nt siRNA), fixed and stained for HPDL (green), actin (red) and nuclei (blue). Images were collected with a Nikon A1 confocal microscope. Bar, 20 μm. HPDL intensity was quantified with ImageJ. (P) MDA-MB-231 cells transfected with siRNA targeting HPD (HPD siRNA) or [file pbio.3002406.s008.tif]

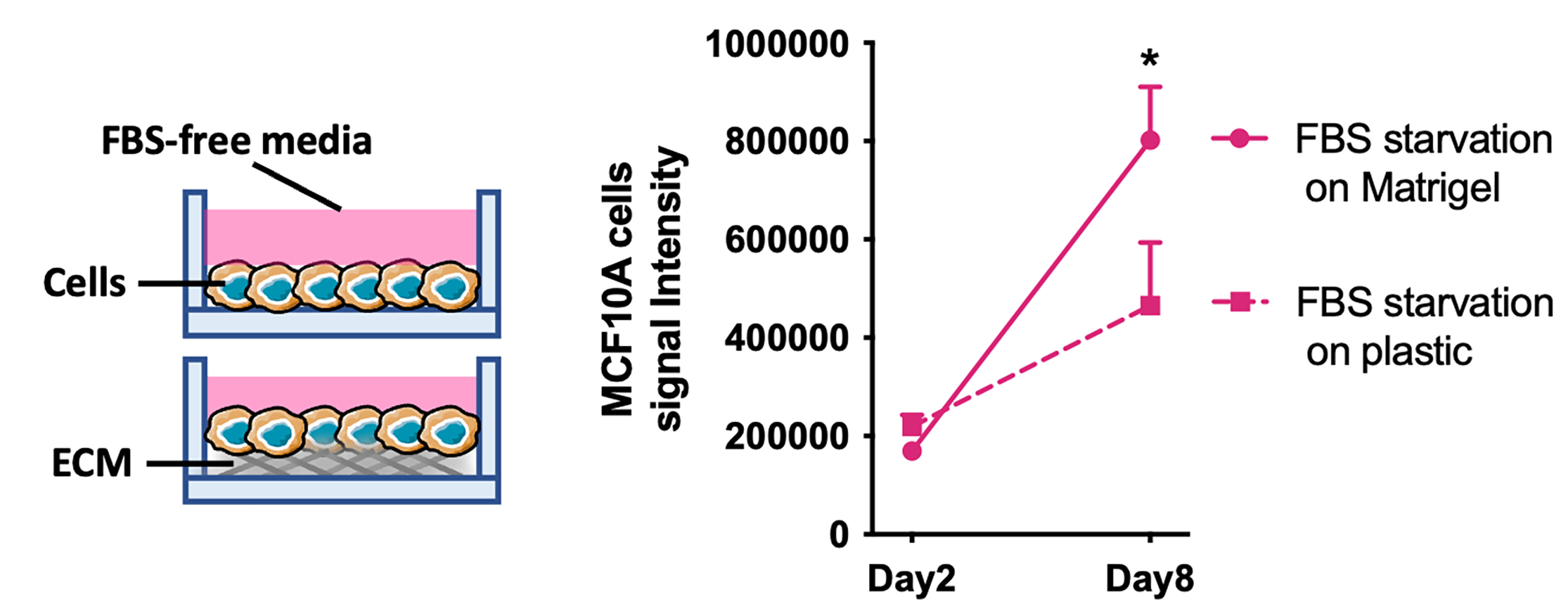

Supplement: S9 Fig — MCF10A cells were seeded on plastic or 3 mg/ml Matrigel for 8 days under serum (FBS) starvation, fixed, stained with DRAQ5 and imaged with a Licor Odyssey system. Signal intensity was calculated by Image Studio Lite software. Values are mean ± SEM from 3 independent experiments. *p = 0.0230 two-way ANOVA, Tukey’s multiple comparisons test. All the raw data associated with this figure are available in S17 Data. (TIF) [file pbio.3002406.s009.tif]

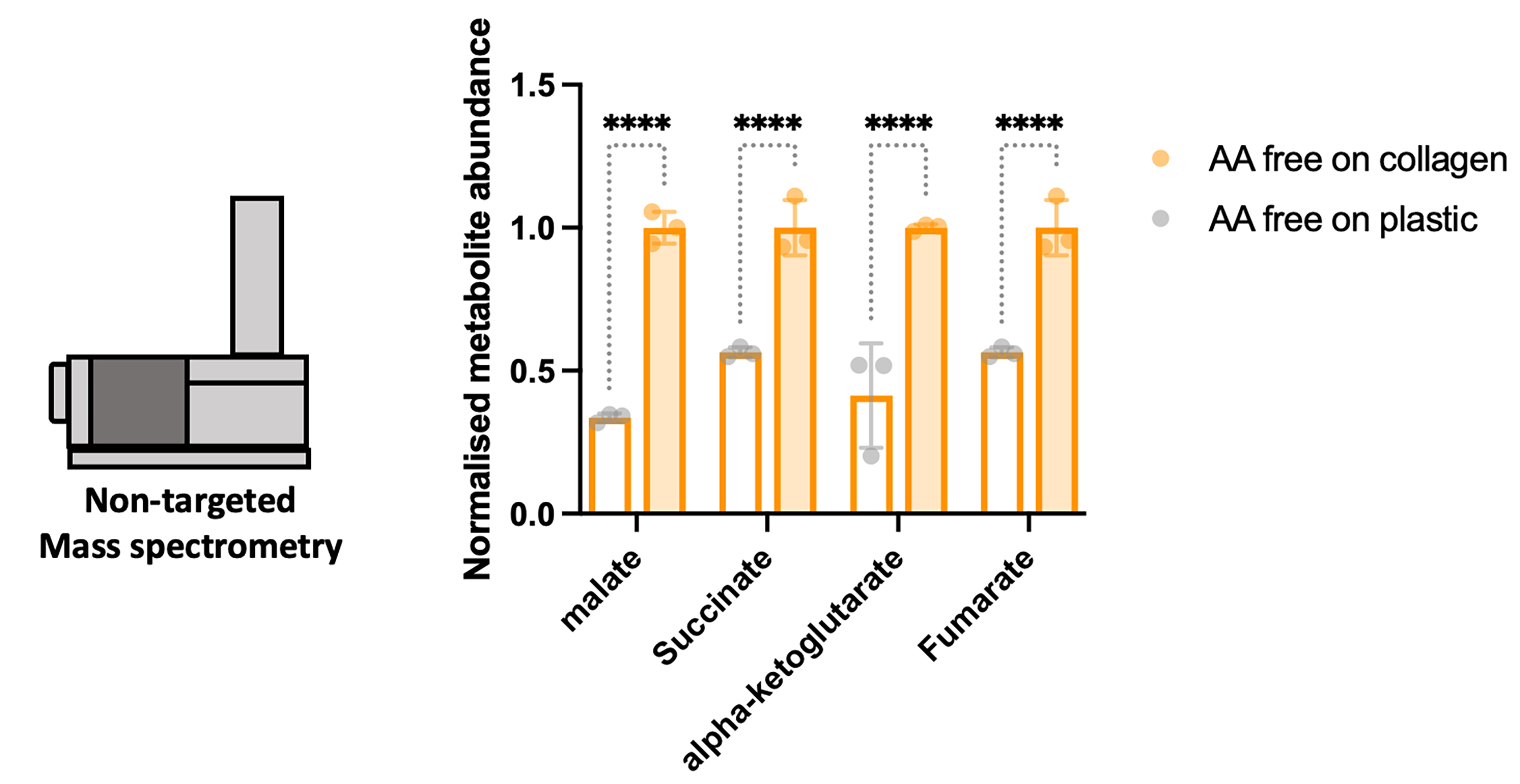

Supplement: S10 Fig — MDA-MB-231 cells were plated on plastic or 2 mg/ml collagen I for 6 days in amino acid-free media. Metabolites were extracted and quantified by non-targeted mass spectrometry. ****p < 0.0001 two-way ANOVA, Tukey’s multiple comparisons test. All the raw data associated with this figure are available in S18 Data. (TIF) [file pbio.3002406.s010.tif]

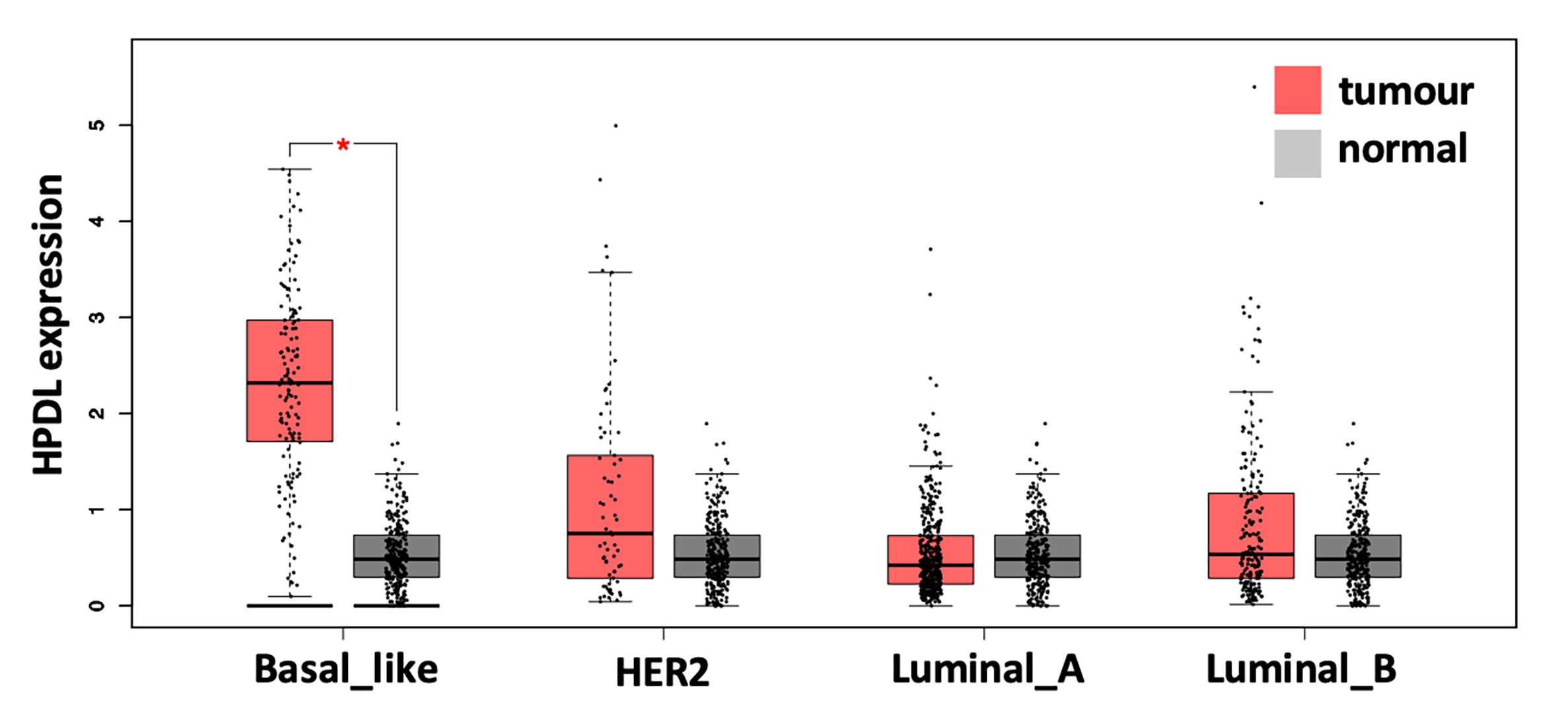

Supplement: S11 Fig — HPDL expression in human tumours and normal tissues, stratified by breast cancer subtypes. Data were obtained from GEPIA 2 (http://gepia2.cancer-pku.cn), *p < 0.01. (TIF) [file pbio.3002406.s011.tif]

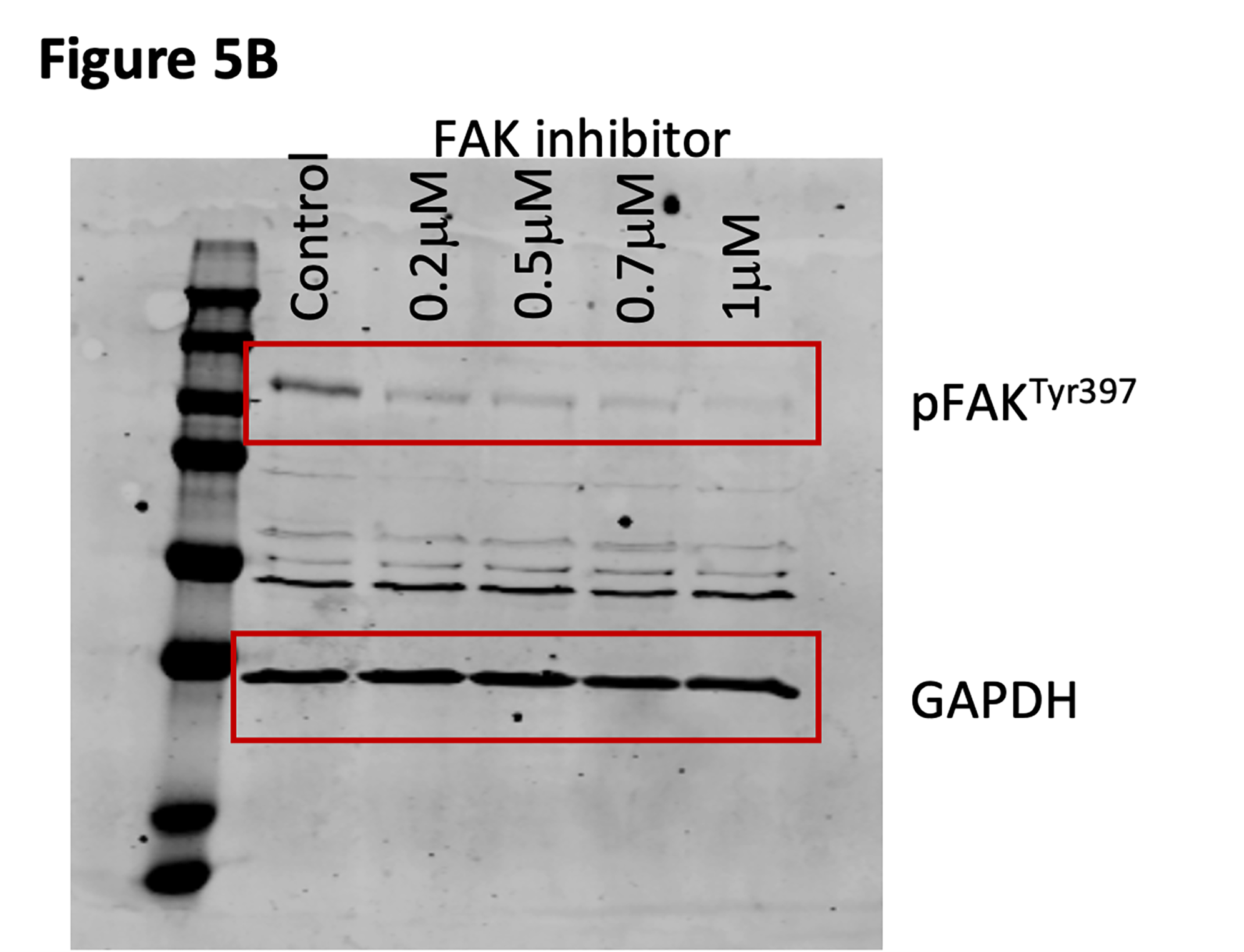

Supplement: S1 Raw Image — (TIF) [file pbio.3002406.s012.tif]

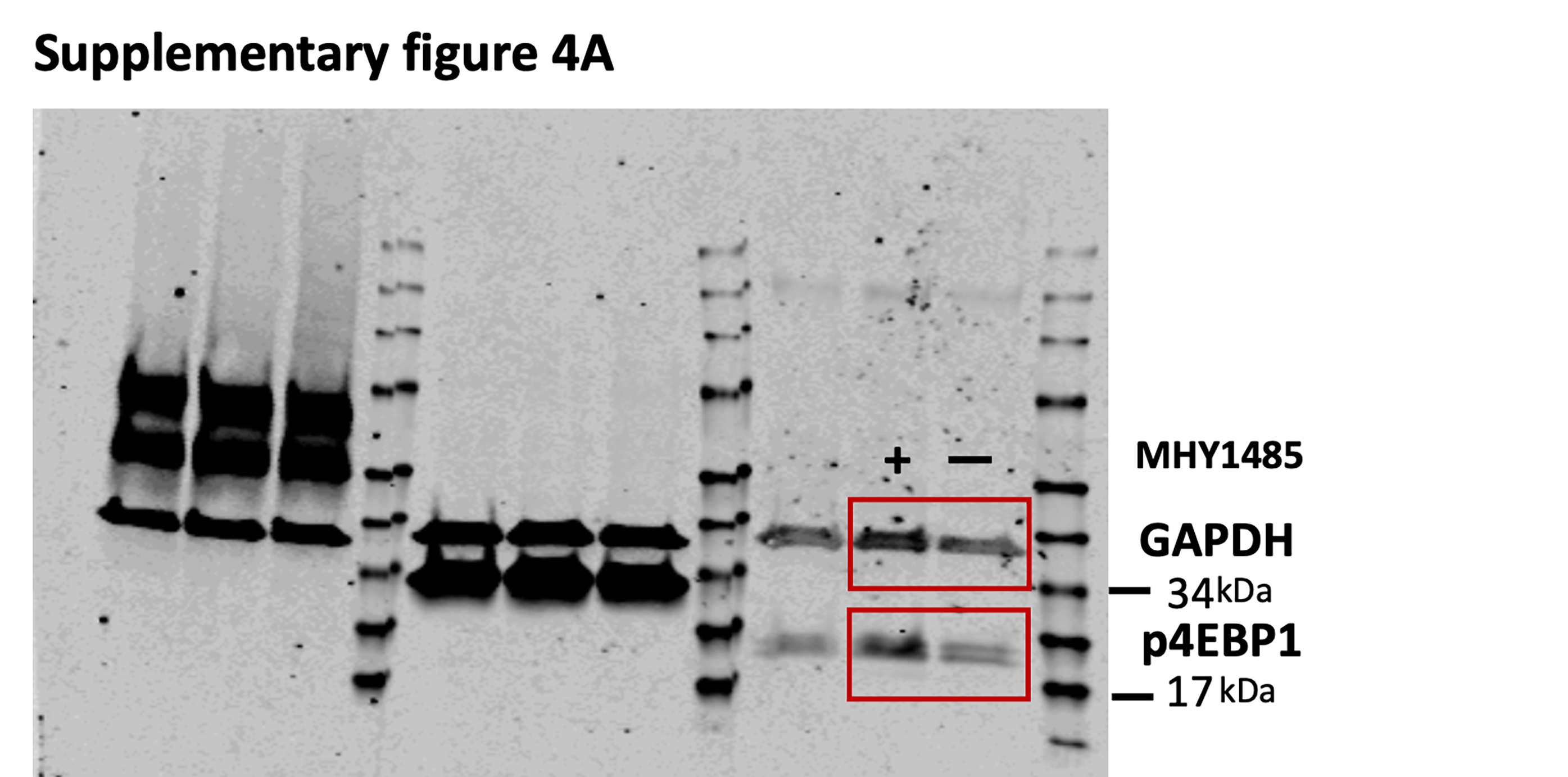

Supplement: S2 Raw Image — (TIF) [file pbio.3002406.s013.tif]

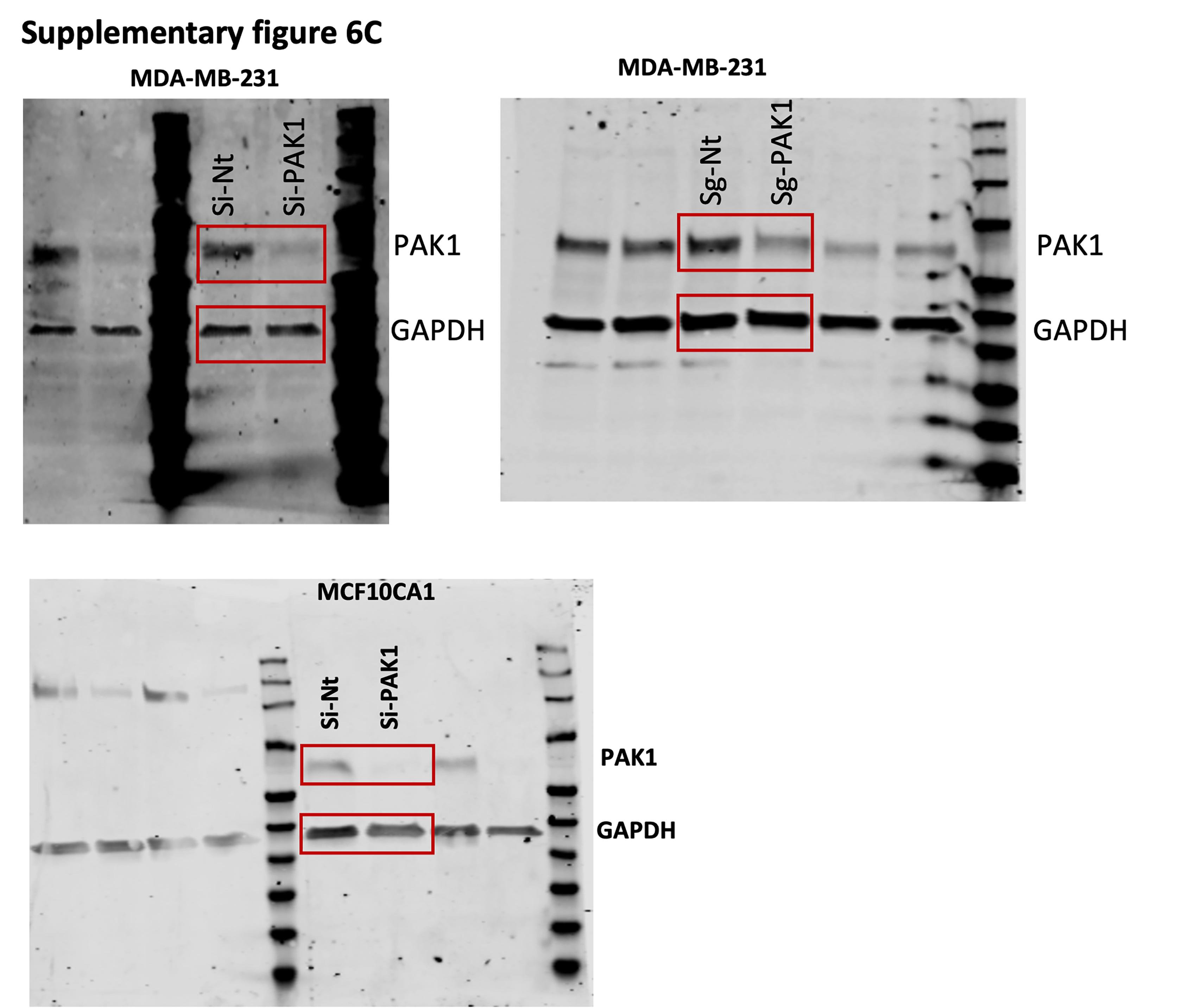

Supplement: S3 Raw Image — (TIF) [file pbio.3002406.s014.tif]

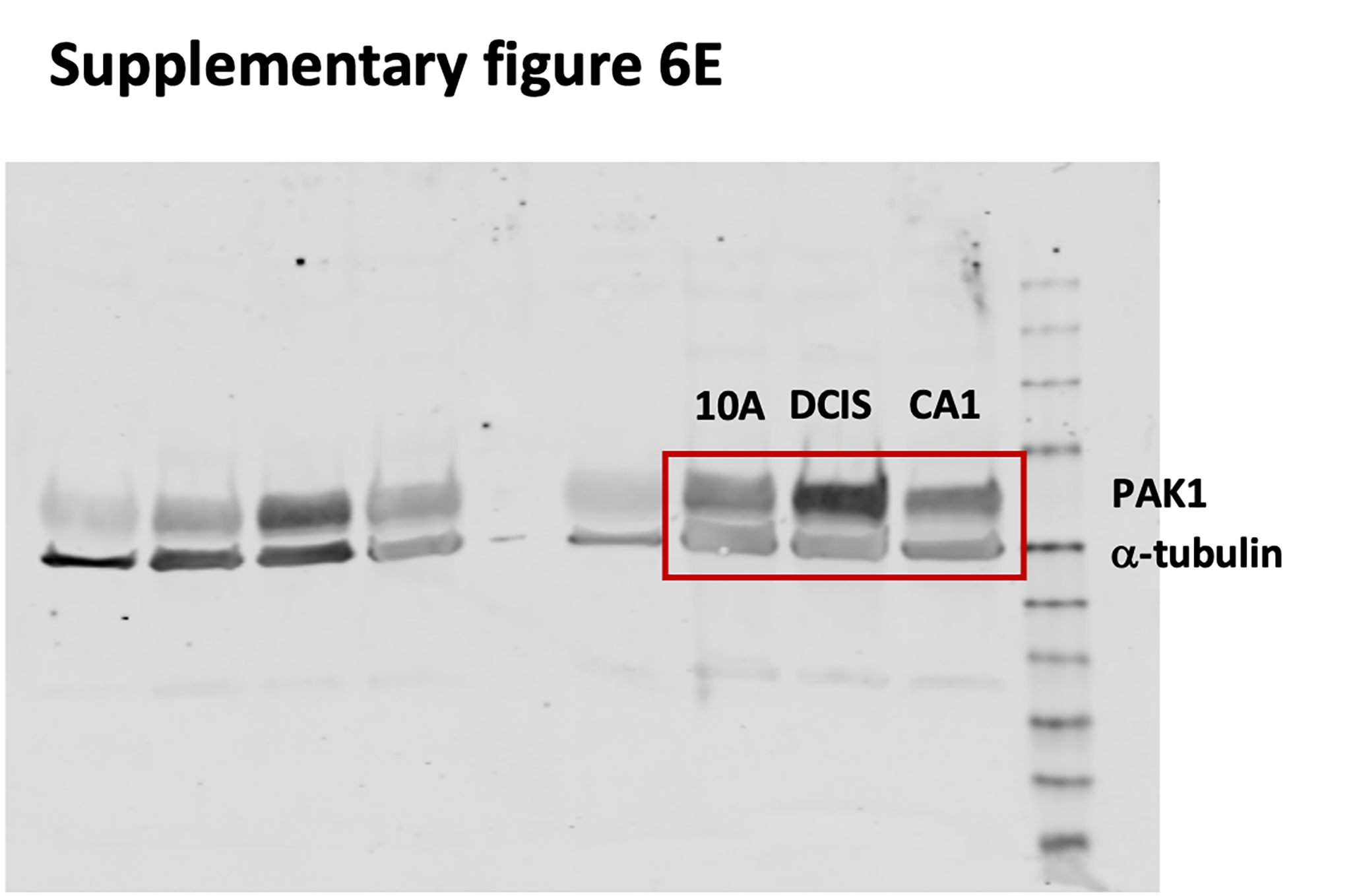

Supplement: S4 Raw Image — (TIF) [file pbio.3002406.s015.tif]
