# Supplementary material for: The extracellular matrix supports breast cancer cell growth under amino acid starvation by promoting tyrosine catabolism
Source: PLoS Biol. 2024 Jan 16;22(1):e3002406. doi: 10.1371/journal.pbio.3002406 (PMC10791009; doi:10.1371/journal.pbio.3002406)
Supplement: Attachment — Submitted filename: 132308_1_rebuttal_2620980_rrbd8l.pdf [file pbio.3002406.s034.pdf]

Revision Plan

Manuscript number: RC-2022-01773
Corresponding author(s): Elena, Rainero

1. General Statements [optional]

We would like to thank all the reviewers for their positive evaluations of our work and constructive comments, in particular for highlighting that our work "provides new insight into cancer metabolism knowledge", is "conceptually interesting and experimentally well performed" and "the findings presented here will be very interesting to a broad range of researchers, including the cancer, metabolism and wider cell biology communities".

2. Description of the planned revisions

Reviewer #1

Since the authors performed their experiments on invasive breast and pancreatic cancers and it has been noted that stress conditions could promote the escape of cancer cells from the site of origin (e.g., Jimenez and Goding, Cell Metabolism 2018; Manzano et al, EMBO Reports 2020), it would be interesting to evaluate how ECM internalization could have a role in sustaining the invasive abilities of cancer cells under amino acid starvation. Which is the impact of the inhibition of macropinocytosis and tyrosine catabolism on cell invasion? The authors could evaluate this aspect by in vitro 2D and 3D analysis.

This is a very important point, and we are planning to investigate this by using:

- 2D single cell migration assays on cell-derived matrices (we have extensively used this system to characterise invasive cell migration, Rainero et al., 2015; Rainero et al., 2012)
- 3D spheroids assays, to assess collective/3D cell invasion through collagen I and matrigel mixtures.

Both experiments will be performed under amino acid starvation, in the presence of pharmacological inhibitors and siRNAs targeting macropinocytosis (FRAX597, PAK1) and tyrosine catabolism (Nitisinone, HPDL). Preliminary data in rev_fig.1 suggest that both FRAX597 and Nitisinone reduce cell invasiveness.

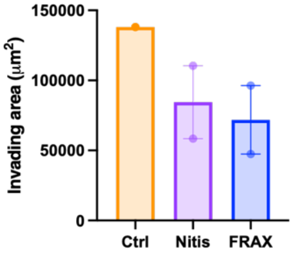

Rev_fig.1: PAK and HPDL inhibition reduced cell invasion. MDA-MB-231 spheroids were generated by the hanging drop method, embedded in 3mg/ml collagen I and Geltrex (50:50) mixture under amino acid starvation and treated with 40μM Nitisinone or 3μM FRAX597 for 2 days. N=1 independent experiment.

Reviewer #2

2. To importantly improve the potential impact of this manuscript, I suggest to add in vivo data using either syngenic mice model of breast cancer or xenografted human breast cancer cells in

nude mice. What would be the impact of micropinocytosis and tyrosine catabolism inhibition on cancer growth, in vivo, should be demonstrated? If possible, it may be interesting to demonstrate that this micropinocytosis may interfere with cancer evolution toward a metastatic phenotype using, for example, the PyMT-MMTV mice model of breast cancer development?

We will perform orthotopic mammary fat pad injections in immunocompetent mice, to monitor primary tumour growth and localised invasion in the presence of Nitisinone or vehicle control. PyMT-driven breast cancer cells have been generated in the Blyth lab, from FVB-pure MMTV-PyMT mice and we have preliminary data indicating that these cells are able to internalise ECM and grow under starvation in an ECM-dependent manner. Prior to performing any in vivo work, we will perform further in vitro experiments to confirm the role of tyrosine catabolism in these cells. Nitisinone is an FDA-approved drug that has already been used in mouse models. Blood tyrosine levels can be measured to assess tyrosine catabolism inhibition by Nitisinone. These experiments will be conducted in collaboration with the Blyth lab at the CRUK Beatson Institute in Glasgow.

3. Data obtained using cancer cells with different metastatic property suggest that the ability to use ECM to compensate for soluble nutrient starvation is acquired during cancer progression. To further demonstrate that it is the case, would it be possible that non metastatic breast cancer cells are not able to perform micropinocytosis? Is PAK1 overexpressed with increase cancer cells metastatic ability, without affecting invasive capacity in 3D spheroids?

To address these points, we have started to measure PAK1 expression across the MCF10 series of cell lines, where MCF10A are non-transformed mammary epithelial cells, MCF10A-DCIS are ductal carcinoma in situ cells and MCF10CA1 are metastatic breast cancer cells. Our preliminary data show that there is no upregulation of PAK1 expression in the metastatic cells compared to non-transformed or non-invasive cancer cells. This suggest that the over-expression of PAK1 might not be a valuable strategy to address this point.

In addition, we found that collagen I uptake was upregulated in MCF10CA1 compared to MCF10A and MCF10A-DCIS. We will corroborate our preliminary data by quantifying collagen I and cell-derived matrix internalisation across the 3 cell lines.

What would be the efficacy to promote the ECM-dependent growth under starvation following a mTORC1 in non-invasive cancer cells?

We will measure the growth of MCF10A and MCF10A-DCIS on ECM under starvation in the presence of the mTOR activator MHY1485. Western blot analysis of downstream targets of mTORC1 will confirm the extent of mTOR activation.

4. The discrepancy of cancer cells proliferation under starvation condition between plastic and ECM-based supports could be explained by the massive difference of support rigidity. This is also probably the case between CDM made by normal fibroblast and CAF. It brings the question of studying the role of matrix stiffness in regard to micropinocytosis-dependent cancer cells growth. It would also explain why this process is link to aggressive cancer cell behaviour, as ECM goes stiffer with time in cancer development. It may not be the case, but the demonstration that mechanical cues from the ECM could regulate the micropinocytosis-dependent cancer cells growth under amino acid starvation could bring additional value to the manuscript.

We will use 2 experimental approaches to address the effect of different stiffness in ECM-dependent cell growth:

A. Polyacrylamide hydrogels coated with different ECM components.

B. Collagen I gels in which the stiffness is modified by Ribose treatment (this approach has been published by the Parson's lab). Our preliminary data confirmed that ribose cross-linking increased YAP nuclear localisation and collagen I can still be internalised under these conditions.

We will assess ECM endocytosis and cell growth under starvation conditions (using EdU incorporation in conjunction with A and high throughput imaging with B).

7. In SF 3A-C, it is shown that ECM does not affect caspase-dependent cell death under AA starvation. Did you considered a non-caspase dependent cell death that may be triggered by AA starvation?

We will complement the caspase 3/7 data by performing propidium iodide (PI) staining, to detect all forms of cell death. Preliminary data indicate that, consistent with our cas3/7 data, amino acid starvation promotes cell death, but the presence of the ECM doesn't affect the percentage of PI positive cells (rev_fig.2), corroborating our conclusions that the ECM modulates cell proliferation and not cell death. We will complete these experiments in both MDA-MB-231 and MCF10CA1 cells and will include them in figure S3.

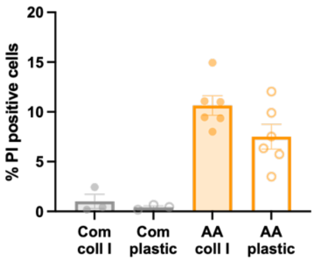

Rev_fig.2: The presence of ECM did not affect cell death under starvation. MDA-MB-231 were seeded on plastic or 2mg/ml collagen I (coll I) for 3 days in complete media (Com) or amino acid-free media (AA). Cells were stained with propidium iodide (PI) and Hoechst 33342, imaged by ImageXpress micro and analysed with MetaXpress software. N=1 independent experiment.

8. In fig 5, it is shown that inhibition of Focal Adhesion Kinase (FAK) does not impair the ECM-dependent rescue of cancer cell growth under starvation. To further decipher the concept of adhesion dependent signalling, maybe the authors could also inhibit the Src kinase or ITG-beta1 activation?

Integrin β1 is also required for ECM internalisation (our unpublished data, manuscript in preparation), therefore interfering with integrin function would make the interpretation of the data quite complex. As suggested by the reviewer, we will use the Src inhibitor PP2, which has been extensively used in the literature in MDA-MB-231 cells. Preliminary data indicate that, despite significantly reducing cell proliferation in complete media, Src inhibition does not affect cell growth on collagen I under amino acid starvation (rev_fig.3), consistent with our FAK inhibitor data. In addition, we performed a kinome and phosphatome screen to identify regulators of ECM endocytosis and Src was not among the hits significantly affecting this process (this work is part of a manuscript in preparation, therefore will not be included in this publication). We will complete these experiments on both collagen I and cell-derived matrices and will include them in figure 5.

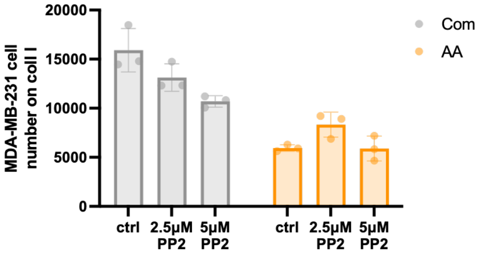

Rev_fig.3: Src inhibition reduced cell growth in complete media but not under amino acid starvation. MDA-MB-231 were seeded on plastic or 2mg/ml collagen I (coll I) for 4 days in complete media (Com) or amino acid-free media (AA), in the presence of the indicated concentrations on PP2 or vehicle control. Cells were fixed, stained with Hoechst 33342, imaged by ImageXpress micro and analysed with MetaXpress software. N=1 independent experiment.

Reviewer #3

- The evaluation of uptake pathways is very interesting. The focus on macropinocytosis is not entirely justified in our opinion looking at FigS4A. Caveolin1/2 and DNM1/3 seem to have strongest effect on uptake of Matrigel and not PAK1? Statements like "Since our data indicate that macropinocytosis is the main pathway controlling ECM endocytosis..." are not justified nor are they really needed in our opinion. Several pathways can be implicated in passive uptake.

We have now removed the statement, as suggested by the reviewer. In addition, we will assess CDM uptake upon caveolin 1/2 and DNM 2/3 knock-down, to test whether the effects are matrigel specific.

-Was the fetal bovine serum (FBS) and Horse Serum (HS) the authors use in their experiments tested for ECM components? The authors mention that the FBS for MDA231 cells was dialysed but not the HS.

Horse serum (HS) was used at a much lower concentration that FBS in our cell proliferation experiments (2.5% HS in CA1 cells compared to 10% FBS). We will characterise both sera components by mass spectrometry analysis, in collaboration with Dr Collins, biOMICS Facility, University of Sheffield.

-Please can the authors provide experimental data directly comparing NF-CAM versus CAF-CDM on the same graph (Figure 1D-E).

In the experiments included in the manuscript, the two matrices were generated independently, and we don't feel it is appropriate to combine the results in the same graph. We are now repeating these experiments by generating both matrices in the same plates, so that we can present the data in the same graph.

3. Description of the revisions that have already been incorporated in the transferred manuscript

Reviewer #1

To strengthen the paper and give a stronger significance in terms of clinical translatability, it could be useful to implement the analysis of breast and pancreatic patients by publicly dataset evaluating for example free survival, disease free survival, overall survival and metastasis free survival.

We have now included in the manuscript new data in figure 6 O-R showing that high HPDL expression correlates with reduces overall survival, distant metastasis-free survival, relapse-free

survival and palliative performance scale in breast cancer patients. In response to other reviewers' comments, we have removed the pancreatic cancer data from our manuscript.

The text and the figures are clear and accurate. The references cited support the hypothesis, rightly introduce the results and are appropriate for the discussion. However, the paragraph relative to figure 4 is a little confusing. Changing the order of the description of the results could be useful.

We apologise for the lack of clarity in this section. We have now re-organised the data both in the figure and in the result section, to describe the findings in a more logical way.

Reviewer #2

1. Despite the reviewer proposition, I believe that the additional experiments using the PDAC cancer cell does not improve the quality of the manuscript. Instead, it brings confusion to me, since the relative addition is minor compare to what is demonstrated using breast cancer cells.

We have decided to remove the pancreatic cancer cell data from the manuscript.

5 It has been demonstrated that matrix rigidity regulates glutaminolysis in breast cancer, resulting in aspartate production and cancer cells proliferation. Is asparate production increase by micropinocytosis? Could you rescue cancer cells growth by aspartate supplementation?

Our metabolomics experiments were performed under amino acid starvation; therefore, glutamine was not present in the media. Nor glutaminolysis intermediates nor aspartate were upregulated on ECM compared to plastic in our dataset, suggesting that aspartate might not be involved in this system. We added this point in the discussion. However, glutamine, glutamate and aspartate were found to be upregulated on collagen I compared to plastic in complete media, where the most enriched pathway was alanine, aspartate and glutamate metabolism. Future work will address the role of the ECM in supporting cancer cell metabolism in the absence of nutrient starvation.

6. *Data presented in Fig 1 and SF1 show that breast cancer cell lines growth in a comparable manner either they are cultured on plastic or on 3D ECM substrates in complete media. Again, on thick 3D substrates, in which the stiffness is lower compared to plastic, I would have thought that cancer cells would have grown slower. Could you please discuss this finding in regard to the literature*?

Our experiments in full media were performed in the presence of dialysed serum, to represent a better control for the starvation conditions. This is consistent with a vast body of literature assessing nutrient starvation conditions in the presence of dialysed serum. This could explain the discrepancy between ours and published results. We have addressed this point in the discussion.

If you have the capacity to do so in your lab or in collaboration, would it be possible to measure the exact stiffness of the different matrix you use in this manuscript? Or using hydrogel, would it be possible to study the role of matrix stiffness in the ECM-dependent cancer cells growth under AA starvation? I would understand that this may be out of the scope of the present manuscript, but I again believe that such finding would reinforce the manuscript.

We don't have the capacity to measure the stiffness in our lab, however NF-CDM and CAF-CDM, generated by the same cells and using the same protocol, have been previously measured at ~0.4kPa and ~0.8 kPa, respectively (Hernandez-Fernaud et al., 2017). We have now included

this in the paper. As mentioned in response to point 4, we will use hydrogels and ribose-treated collagen I gels to directly test the effect of matrix stiffness on ECM-dependent cell growth under nutrient starvation.

9. Minor comment, in F1B, it is written "AA free starvation" while in every others legend, it is written "AA starvation". I believe the "free" should be removed.

We apologise for this mistake; we have now removed “free” from the legend.

Reviewer #3

-The ECM mediated increase of cell growth under amino acid (AA) starvation is nicely shown In Fig.1 but the authors should include the full medium data from figure S1 in the graphs of Fig. 1 to enable the reader to evaluate the magnitude of rescue effect of the ECM components. The values should also be included in the results text.

We have now moved all the complete media data into the main figures and highlighted the extent of the rescue in the result section.

Also the authors only glutamine starve in Fig1&2 and then don't mention it again can the authors please include a sentence to explain why this experiment was dropped.

As now highlighted in the result section, we focused on the amino acid starvation as it resulted in the strongest difference between normal and cancer cells. On the one hand, also non-invasive breast cancer cells can use ECM (namely matrigel) to grow under glutamine starvation, while this is not the case under amino acid starvation. On the other hand, only CAF-CDM, but not normal-CDM, could rescue cell growth under amino acid starvation. We reasoned that this condition was more likely to identify cancer-specific phenotypes.

-The pancreatic cancer data currently feels a bit like an afterthought. We suggest to remove this data from the manuscript. If this data is included we suggest the authors should expand this section and repeat key experiments of earlier figures.

We have now removed these data from the manuscript, as this was also the suggestion of reviewer #2.

-Please can the authors give more insight to the use of 25% Plasmax to mimic starved tumor microenvironment. Is there previous research that suggests the nutrient values are representative of TME?

Apologies for not clarifying this in the initial submission, the rationale behind this choice is based on the observation that, in pancreatic cancers, nutrients were shown to be depleted between 50-75% (Kamphorst et al., 2015). We have now stated this in the result section.

-Fig3E Can the authors please include example images of the pS6 staining in the supplementary figures and explain "mTOR endosomal index" in figure legend.

We have now included the representative images (new figure 3E) and we have described how the mTOR endosomal index was calculated both in the figure legend and in the method section.

- The PAK1 expression level blots in the knockdown experiments should be quantified from N=3.

We have now included the quantification of the western blots in the new supplementary figure 5.

-What is the FA index in Fig.5, explain how it is calculated. Why not use FA size alone?

We have now defined this is the method section. We haven’t used FA size alone, as this measure can be affected by cell size. If a cell is bigger, the overall FA size will be bigger, but this doesn’t necessarily reflect a change in adhesions.

-Can the authors please use paragraphs on page 9 to improve readability.
We apologise for overlooking this, we have now used paragraphs in this section.

4. Description of analyses that authors prefer not to carry out

Reviewer #3

- The authors use FAK inhibition to evaluate the effect of focal adhesion signalling on their phenotypes and conclude that there is no connection between the observed increase of cell proliferation in presence of ECM and adhesion signalling. To make this assessment the authors need at the very least to show that their FAK inhibitor treatment at the used concentration results in changes in focal adhesions and the associated force transduction.

In the result section, we are including a western blot analysis showing that the concentration of FAK inhibitor used in sufficient to significantly reduced FAK auto-phosphorylation (figure 5B). Based on published evidence (Horton et al., 2016), FAK inhibition does not affect focal adhesion formation, but only the phosphorylation events. Therefore, we don't think that we will be able to detect changes in focal adhesions regardless of the concentration of the inhibitor we use. To strengthen the observation that ECM-dependent cell growth in independent from adhesion signalling, as suggested by reviewer #2, we will use the Src inhibitor PP2, which has been extensively used in the literature in MDA-MB-231 cells. Preliminary data indicate that, despite significantly reducing cell proliferation in complete media, Src inhibition does not affect cell growth on collagen I under amino acid starvation (rev_fig.3 above), consistent with our FAK inhibitor data. We will complete these experiments on both collagen I and cell-derived matrices and will include them in figure 5.

-Can the authors include a negative control for the mTORC1 localisation in Fig.3 (such as use of rapamycin/Torin)?

Amino acid starvation is the gold-standard control for mTORC1 lysosomal targeting, as described in a variety of publications, including Manifava et al., 2016; Meng et al., 2021; Averous et al., 2014. In addition, Torin 1 treatment has been shown to result in a significant accumulation of mTOR on lysosomes compared with untreated cells (Settembre et al., 2012). Consistent with this, we looked at mTOR localisation in the presence of Rapamycin and we did not detect any reduction in lysosomal targeting.
