# Supplementary material for: The extracellular matrix supports breast cancer cell growth under amino acid starvation by promoting tyrosine catabolism
Source: PLoS Biol. 2024 Jan 16;22(1):e3002406. doi: 10.1371/journal.pbio.3002406 (PMC10791009; doi:10.1371/journal.pbio.3002406)
Supplement: Attachment — Submitted filename: Response_to_reviewers_Nazemi.pdf [file pbio.3002406.s036.pdf]

We would like to thank all the reviewers for their positive evaluations of our work and constructive comments. We have addressed all the concerns raised and modified the manuscript accordingly (all the changes are highlighted in blue).

Reviewer #1
<u>Major comment</u>:
Since the authors performed their experiments on invasive breast and pancreatic cancers and it has been noted that stress conditions could promote the escape of cancer cells from the site of origin (e.g., Jimenez and Goding, Cell Metabolism 2018; Manzano et al, EMBO Reports 2020), it would be interesting to evaluate how ECM internalization could have a role in sustaining the invasive abilities of cancer cells under amino acid starvation. Which is the impact of the inhibition of macropinocytosis and tyrosine catabolism on cell invasion? The authors could evaluate this aspect by in vitro 2D and 3D analysis.

We thank the reviewer for raising this point, we feel this part adds impact to our manuscript. To investigate the role of macropinocytosis and tyrosine catabolism on cell invasion, we measured pseudopod elongation during cell migration on CAF cell-derived matrix CDM) under amino acid starvation. Extensive literature indicate that pseudopod elongation is indicative of invasive behaviour (Rainero et a., 2012; Rainero et al., 2015). Interestingly, both siRNA-mediated knockdown and pharmacological inhibition of HPDL and PAK1 resulted in a statistically significant reduction in pseudopod elongation (**fig 8A-C**). Moreover, HPDL and PAK1 inhibition also significantly impaired breast cancer cell invasion using 3D spheroids embedded in ECM under amino acid starvation (**fig 8D-F**).

In addition, to strengthen the paper and give a stronger significance in terms of clinical translatability, it could be useful to implement the analysis of breast and pancreatic patients by publicly dataset evaluating for example free survival, disease free survival, overall survival and metastasis free survival.

We have now included in the manuscript new data in figure 6 O-R showing that high HPDL expression correlates with reduces overall survival, distant metastasis-free survival, relapse-free survival and palliative performance scale in breast cancer patients. In response to other reviewers' comments, we have removed the pancreatic cancer data from our manuscript.

<u>Minor Comment</u>:
The text and the figures are clear and accurate. The references cited support the hypothesis, rightly introduce the results and are appropriate for the discussion. However, the paragraph relative to figure 4 is a little confusing. Changing the order of the description of the results could be useful.

We apologise for the lack of clarity in this section. We have now re-organised the data both in the figure and in the result section, to describe the findings in a more logical way.

Reviewer #2

1. Despite the reviewer proposition, I believe that the additional experiments using the PDAC cancer cell does not improve the quality of the manuscript. Instead, it brings confusion to me, since the relative addition is minor compare to what is demonstrated using breast cancer cells.

We have decided to remove the pancreatic cancer cell data from the manuscript.

2. ***To importantly improve the potential impact of this manuscript, I suggest to add in vivo data using***

***either syngenic mice model of breast cancer or xenografted human breast cancer cells in nude mice. What would be the impact of micropinocytosis and tyrosine catabolism inhibition on cancer growth, in vivo, should be demonstrated? If possible, it may be interesting to demonstrate that this micropinocytosis may interfere with cancer evolution toward a metastatic phenotype using, for example, the PyMT-MMTV mice model of breast cancer development*?**

We performed orthotopic mammary fat pad injections in immunocompetent mice, to monitor primary tumour growth and lung metastasis formation in the presence of Nitisinone or vehicle control. PyMT-driven breast cancer cells have been generated in the Blyth lab, from FVB-pure MMTV-PyMT mice and we showed that these cells were able to internalise ECM in a macropinocytosis-dependent manner and ECM-dependent cell growth required tyrosine catabolism. Despite this, we did not observe any difference in tumour growth or lung metastasis in vivo (**fig rev 1**). It is important to consider that the PyMT model transcriptionally resembles Luminal B breast cancer, while in human tumours HPDL overexpression is mainly observed in triple negative breast cancer, suggesting that the role of tyrosine catabolism might be subtype specific. We feel that it is important to carry out additional work, outside the scope of this manuscript, to characterise the subtype specificity and perform further in vivo characterisation, using different mouse models.

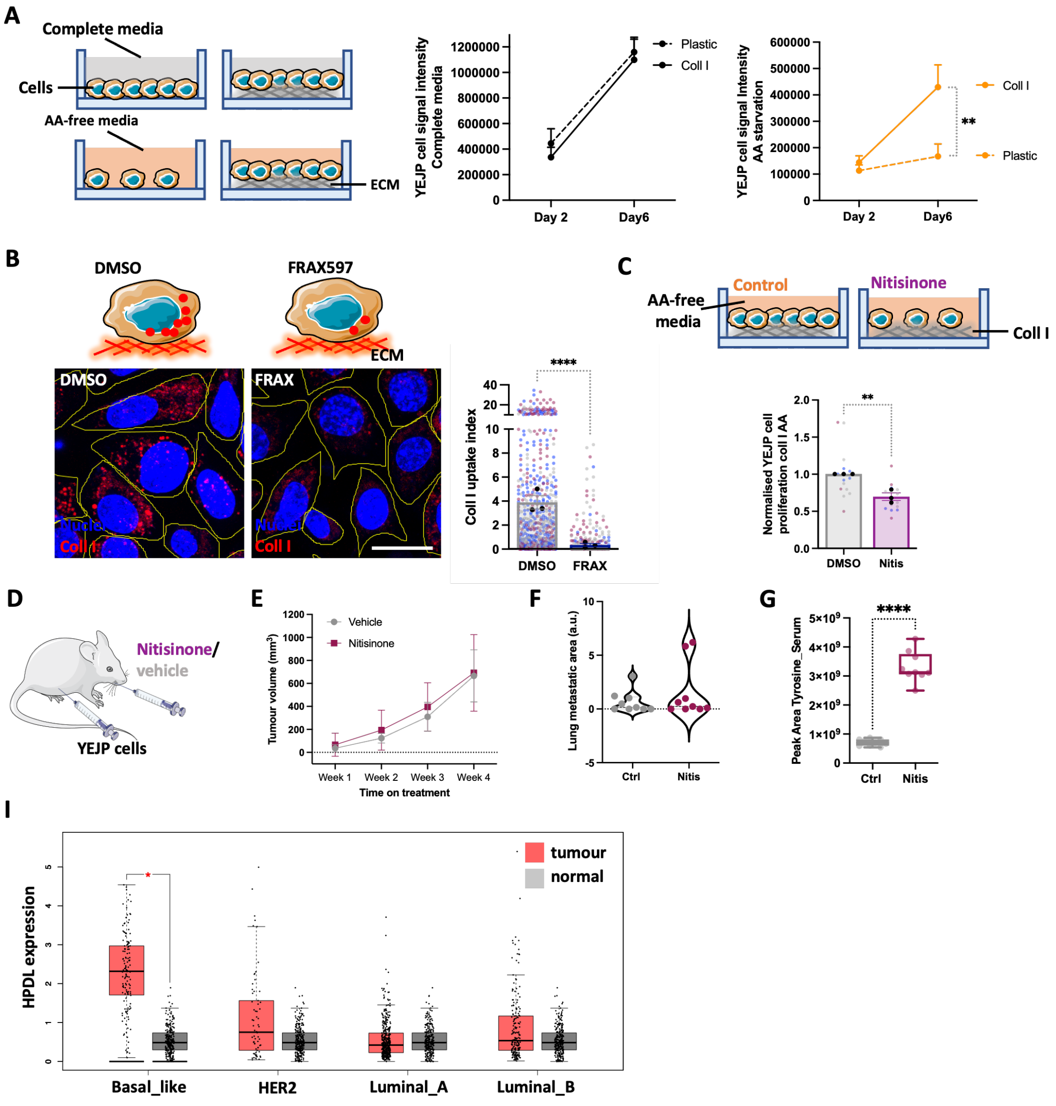

*Fig.rev1. **Nitisinone treatment did not affect tumour growth in vivo**. (**A**) YEJP cells were seeded on plastic or 2mg/ml collagen I (coll I) for 6 days under complete media or amino acid (AA) starvation, fixed, stained with DRAQ5 and imaged with a Licor Odyssey system. Signal intensity was calculated by*

*Image Studio Lite software. Values are mean ± SEM and are representative of 3 independent experiments. **p=0.0019 2way ANOVA, Tukey's multiple comparisons test. (**B**) YEJP cells seeded on pH-rodo labelled 1mg/ml collagen I for 6 hrs, in the presence of 3μM FRAX597 or DMSO control, stained with Hoechst 33342, imaged live with a Nikon A1 confocal microscope and quantified with Image J. Scale bar, 20μm. Values are mean ± SEM and are representative of 3 independent experiments. ****p<0.0001 Mann-Whitney test. (**C**) YEJP cells were seeded 2mg/ml collagen I (coll I) in the presence of 40μM nitisinone (nitis) or DMSO control for 4 days under amino acid (AA) starvation, fixed, stained with DRAQ5 and imaged with a Licor Odyssey system. Signal intensity was calculated by Image Studio Lite software. Values are mean $\pm$ SEM and are representative of 3 independent experiments. **p=0.0014 Mann-Whitney test. (**D**) Schematic, in vivo experiment. YEJP mammary tumour cells were orthotopically transplanted into 20 mice, divided into two groups with 10 mice receiving Nitisinone (8mg/kg of body weight every other day by oral gavage), and 10 mice receiving vehicle. (**E**) Weekly average of n=9 Vehicle (blue line) and n=9 Nitisinone (red line) tumours ±SD. One mouse from each group did not develop tumours and was excluded from analysis. (**F**) Violin plot depicting metastatic area of lungs scored by H&E and validated by PyMT staining in n=9 Ctrl (blue) or n=9 Nitisinone (red). (H) Serum tyrosine levels was measured in n=9 Vehicle-treated mice (blue) and n=9 of Nitisinone-treated mice (red) by mass spectrometry. ****p<0.001. (**I**) HPDL expression in human tumours and normal tissues, stratified by breast cancer subtypes. Data were obtained from GEPIA 2 (http://gepia2.cancer-pku.cn).*

3. Data obtained using cancer cells with different metastatic property suggest that the ability to use ECM to compensate for soluble nutrient starvation is acquired during cancer progression. To further demonstrate that it is the case, would it be possible that non metastatic breast cancer cells are not able to perform micropinocytosis? Is PAK1 overexpressed with increase cancer cells metastatic ability, without affecting invasive capacity in 3D spheroids? What would be the efficacy to promote the ECM-dependent growth under starvation following activation of mTORC1 in non-invasive cancer cells?

We thank the reviewer for raising this point. In the new version of the manuscript, we show that indeed ECM internalisation is strongly increased in metastatic breast cancer cells, compared to non-invasive breast cancer cells and non-transformed mammary epithelial cells (**fig 4A,B**). However, this phenomenon is not due to PAK1 over-expression, as we did not detect any significant changes in PAK1 protein levels across the 3 cell lines (**fig S6E**).

To establish whether the activation of mTOR is sufficient to promote the growth of non-transformed and non-invasive breast cancer cells, we treated MCF10A and MCF10A-DCIS cells with the mTOR activator MHY1485. Consistent with the literature (Choi et al., 2012), we observed a small but statistically significant increase in the phosphorylation of the mTOR target 4EBP1. However, treatment with the mTOR activation did not affect cell growth on collagen I under amino acid starvation (**fig S4**).

4. The discrepancy of cancer cells proliferation under starvation condition between plastic and ECM-based supports could be explained by the massive difference of support rigidity. This is also probably the case between CDM made by normal fibroblast and CAF. It brings the question of studying the role of matrix stiffness in regard to micropinocytosis-dependent cancer cells growth. It would also explain why this process is link to aggressive cancer cell behaviour, as ECM goes stiffer with time in cancer development. It may not be the case, but the demonstration that mechanical cues from the ECM could regulate the micropinocytosis-dependent cancer cells growth under amino acid starvation could bring additional value to the manuscript.

We agree with the reviewer that it is important to consider the implication of matrix rigidity in ECM-dependent cell growth. To address this, we used 2 complementary approaches: collagen I cross-linking by non-enzymatic glycation and polyacrylamide hydrogels of different stiffness. Collagen I stiffness can be increased by non-enzymatic glycation induce by ribose treatment (Pfisterer et al., 2021). While untreated collagen I gels have a stiffness of ~0.2 kPa, 200mM ribose increases it to ~0.8 kPa (Roy et al., 2010; Mason et al., 2013). We found that ribose treatment did not significantly affect collagen I endocytosis and, while it resulted in a small increase in cell growth in complete media, it did not change cell proliferation under amino acid starvation (**fig S7A,B**). Similarly, higher stiffness correlated with slightly reduced cell growth under amino acid starvation (**fig S7C**).

5. ***Along with this, it has been demonstrated that matrix rigidity regulates glutaminolysis in breast cancer, resulting in aspartate production and cancer cells proliferation. Is asparate production increase by micropinocytosis? Could you rescue cancer cells growth by aspartate supplementation*?**

Our metabolomics experiments were performed under amino acid starvation; therefore, glutamine was not present in the media. Nor glutaminolysis intermediates nor aspartate were upregulated on ECM compared to plastic in our datasets, suggesting that aspartate might not be involved in this system. We added this point in the discussion. However, glutamine, glutamate and aspartate were found to be upregulated on collagen I compared to plastic in complete media, where the most enriched pathway was alanine, aspartate and glutamate metabolism. Future work will address the role of the ECM in supporting cancer cell metabolism in the absence of nutrient starvation.

6. ***Data presented in Fig 1 and SF1 show that breast cancer cell lines growth in a comparable manner either they are cultured on plastic or on 3D ECM substrates in complete media. Again, on thick 3D substrates, in which the stiffness is lower compared to plastic, I would have thought that cancer cells would have grown slower. Could you please discuss this finding in regard to the literature*?**

We have addressed this point in the discussion. Our experiments in full media were performed in the presence of dialysed serum, to represent a better control for the starvation conditions. This is consistent with a vast body of literature assessing nutrient starvation conditions in the presence of dialysed serum. This could explain the discrepancy between ours and published results.

If you have the capacity to do so in your lab or in collaboration, would it be possible to measure the exact stiffness of the different matrix you use in this manuscript? Or using hydrogel, would it be possible to study the role of matrix stiffness in the ECM-dependent cancer cells growth under AA starvation? I would understand that this may be out of the scope of the present manuscript, but I again believe that such finding would reinforce the manuscript.

Unfortunately, we don't have the capacity to measure the stiffness in our lab, however NF-CDM and CAF-CDM, generated by the same cells and using the same protocol, have been previously measured at ~0.4kPa and ~0.8 kPa, respectively (Hernandez-Fernaud et al., 2017). We have now included this in the paper. As mentioned in response to point 4, we have used hydrogels to directly test the effect of matrix stiffness on ECM-dependent cell growth under nutrient starvation.

7. ***In SF 3A-C, it is shown that ECM does not affect caspase-dependent cell death under AA starvation. Did you considered a non-caspase dependent cell death that may be triggered by AA starvation*?**

To complement to cleaved caspase 3/7 data, we used propidium iodide (PI) staining, to identify cells undergoing necrosis or late apoptosis (Crowley et al., 2016). Similarly, we found that the presence of collagen I did not significantly affect the proportion of dead cells under amino acid starvation in both MDA-MB-231 and MCF10CA1 breast cancer cells (**fig S3F-J**).

8. In fig 5, it is shown that inhibition of Focal Adhesion Kinase (FAK) does not impair the ECM-dependent rescue of cancer cell growth under starvation. To further decipher the concept of adhesion dependent signalling, maybe the authors could also inhibit the Src kinase or ITG-beta1 activation?

We thank the reviewer for this suggestion. Integrin β1 is also required for ECM internalisation (our unpublished data), therefore interfering with integrin function would make the interpretation of the data quite complex; therefore, we investigated the role of Src in ECM-dependent cell growth. Similarly to our FAK inhibitor data, the treatment of MDA-MB-231 cells with the Src inhibitor PP2 (Sanchez-Bailon et al., 2012) did not oppose ECM-dependent cell growth under amino acid starvation, on both collagen I and CAF-generated CDM. Interestingly, we detected a statistically significant reduction in cell proliferation in complete media (**fig 5G-J**).

9. Minor comment, in F1B, it is written "AA free starvation" while in every others legend, it is written "AA starvation". I believe the "free" should be removed.

We apologise for this mistake; we have now removed “free” from the legend.

Reviewer #3
<u>Major</u>
-The ECM mediated increase of cell growth under amino acid (AA) starvation is nicely shown In Fig.1 but the authors should include the full medium data from figure S1 in the graphs of Fig. 1 to enable the reader to evaluate the magnitude of rescue effect of the ECM components. The values should also be included in the results text.

We apologies for creating confusion. We have now moved all the complete media data into the main figures and highlighted the extent of the rescue in the result section.

Also the authors only glutamine starve in Fig1&2 and then don't mention it again can the authors please include a sentence to explain why this experiment was dropped.

As now highlighted in the result section, we focused on the amino acid starvation as it resulted in the strongest difference between normal and cancer. On the one hand, also non-invasive breast cancer cells can use ECM (namely matrigel) to grow under glutamine starvation, while this is not the case under amino acid starvation. On the other hand, only CAF-CDM, but not normal-CDM, could rescue cell growth under amino acid starvation. We reasoned that this condition was more likely to identify cancer-specific phenotypes.

- The evaluation of uptake pathways is very interesting. The focus on macropinocytosis is not entirely justified in our opinion looking at FigS4A. Caveolin1/2 and DNM1/3 seem to have strongest effect on uptake of Matrigel and not PAK1? Statements like "Since our data indicate that macropinocytosis is the main pathway controlling ECM endocytosis..." are not justified nor are they really needed in our opinion. Several pathways can be implicated in passive uptake.

We thank the reviewer for raising this point and we have now removed the statement, as suggested by the reviewer. In addition, to expand on this, we looked at the contribution of Caveolin 1/2 and DNM2/3 in CDM internalisation and found that both knock-downs significantly reduced CDM uptake, but to a lesser extent than PAK1 KD (**fig S6B,D**).

***- The authors use FAK inhibition to evaluate the effect of focal adhesion signalling on their phenotypes and conclude that there is no connection between the observed increase of cell proliferation in presence of ECM and adhesion signalling. To make this assessment the authors need at the very least to show that their FAK inhibitor treatment at the used concentration results in changes in focal adhesions and the associated force transduction*.**

We agree with both reviewers suggesting to corroborate our findings related to the role of adhesion signalling in ECM-dependent cell growth. In the result section, we showed a western blot analysis showing that the concentration of FAK inhibitor used was sufficient to significantly reduce FAK auto-phosphorylation. Based on published evidence (Horton et al., 2016), FAK inhibition does not affect focal adhesion formation, but only the phosphorylation events. Therefore, we don't think that we will be able to detect changes in focal adhesions regardless of the concentration of the inhibitor we use. To strengthen the observation that ECM-dependent cell growth under starvation is independent from adhesion signalling, as suggested by reviewer #2, we used the Src inhibitor PP2, which has been extensively used in the literature in MDA-MB-231 cells – see response to Reviewer 2's comments.

***-The pancreatic cancer data currently feels a bit like an afterthought. We suggest to remove this data from the manuscript. If this data is included we suggest the authors should expand this section and repeat key experiments of earlier figures*.**

We have now removed these data from the manuscript, as this was also the suggestion of reviewer #2.

***-Was the fetal bovine serum (FBS) and Horse Serum (HS) the authors use in their experiments tested for ECM components? The authors mention that the FBS for MDA231 cells was dialysed but not the HS*.**

We thank the reviewer for raising this point. We now characterised FBS, dialysed FBS and horse sera components by mass spectrometry, in collaboration with Dr Collins, biOMICS Facility, University of Sheffield. Table 2 and 3 contain the serum components identified as ECM or ECM-associated proteins in the matrisome database (https://matrisomedb.org/). Several ECM component have been detected, but given the fact that the sera are also added on cells grown on plastic, we believe that the amount of ECM components present is not enough to support cell growth under starvation.

Minor comments:
-Please can the authors provide experimental data directly comparing NF-CAM versus CAF-CDM on the same graph (Figure 1D-E).

In the experiments included in the first version of the manuscript, the two matrices were generated independently, and we don't feel it is appropriate to combine the results in the same graph. We have now repeated these experiments and confirmed that CAF-CDM, but not NF-CDM, rescued cell proliferation under amino acid starvation (**fig 1G**).

***-Please can the authors give more insight to the use of 25% Plasmax to mimic starved tumor microenvironment. Is there previous research that suggests the nutrient values are representative of TME*?**

Apologies for not clarifying this in the initial submission, the rationale behind this choice is based on the observation that, in pancreatic cancers, nutrients were shown to be depleted between 50-75% (Kamphorst et al., 2015). We have now stated this in the result section.

***-Fig3E Can the authors please include example images of the pS6 staining in the supplementary figures and explain "mTOR endosomal index" in figure legend*.**

We apologise for the lack of clarity. We have now included the representative images (**fig 3E**) and we have described how the mTOR endosomal index was calculated both in the figure legend and in the method section.

***-Can the authors include a negative control for the mTORC1 localisation in Fig.3 (such as use of rapamycin/Torin)*?**

Amino acid starvation is the gold-standard control for mTORC1 lysosomal targeting, as described in a variety of publications, including Manifava et al., 2016; Meng et al., 2021; Averous et al., 2014. In addition, Torin 1 treatment has been shown to result in a significant accumulation of mTOR on lysosomes compared with untreated cells (Settembre et al., 2012). Given these strong pieces of evidence from the literature, we believe this experiment is not necessary.

***- The PAK1 expression level blots in the knockdown experiments should be quantified from N=3*.**

We have not included the quantification of the western blots in **figure S5C**.

***-What is the FA index in Fig.5, explain how it is calculated. Why not use FA size alone*?**

We apologise for the lack of clarity around this concept. We have now defined what the focal adhesion index is in the method section. We haven't used FA size alone, as this measure can be affected by cell size. If a cell is bigger, the overall FA size will be bigger, but this doesn't necessarily reflect a change in adhesions.

***-Can the authors please use paragraphs on page 9 to improve readability*.**

We apologise for overlooking this, we have now used paragraph in this section.
